# Oceanic eddy-induced modifications to air–sea heat and CO_2_ fluxes in the Brazil-Malvinas Confluence

**DOI:** 10.1038/s41598-021-89985-9

**Published:** 2021-05-20

**Authors:** Luciano P. Pezzi, Ronald B. de Souza, Marcelo F. Santini, Arthur J. Miller, Jonas T. Carvalho, Claudia K. Parise, Mario F. Quadro, Eliana B. Rosa, Flavio Justino, Ueslei A. Sutil, Mylene J. Cabrera, Alexander V. Babanin, Joey Voermans, Ernani L. Nascimento, Rita C. M. Alves, Gabriel B. Munchow, Joel Rubert

**Affiliations:** 1 Laboratory of Ocean and Atmosphere Studies (LOA), Earth Observation and Geoinformatics Division (OBT), National Institute for Space Research (INPE), São José dos Campos, SP Brazil; 2 Earth System Numerical Modeling Division, Center for Weather Forecast and Climate Studies (CPTEC), National Institute for Space Research (INPE), Cachoeira Paulista, SP Brazil; 3 Scripps Institution of Oceanography, University of California, San Diego, La Jolla, CA USA; 4 Federal University of Maranhão, São Luís, MA Brazil; 5 Federal Institute of Education, Science and Technology of Santa Catarina, Florianópolis, SC Brazil; 6 Agricultural Engineering Department, Federal University of Viçosa, Viçosa, MG Brazil; 7 Department of Infrastructure Engineering, University of Melbourne, Victoria, Australia; 8 Atmospheric Modeling Group (GruMA), Department of Physics, Federal University of Santa Maria, Santa Maria, RS Brazil; 9 Federal University of Rio Grande do Sul, Porto Alegre, RS Brazil; 10 Southern Space Coordination (COESU), National Institute for Space Research (CRS/INPE) , Santa Maria, RS Brazil

**Keywords:** Physical oceanography, Biochemistry, Environmental sciences, Ocean sciences, Climate sciences, Atmospheric science, Ocean sciences

## Abstract

Sea surface temperature (SST) anomalies caused by a warm core eddy (WCE) in the Southwestern Atlantic Ocean (SWA) rendered a crucial influence on modifying the marine atmospheric boundary layer (MABL). During the first cruise to support the Antarctic Modeling and Observation System (ATMOS) project, a WCE that was shed from the Brazil Current was sampled. Apart from traditional meteorological measurements, we used the Eddy Covariance method to directly measure the ocean–atmosphere sensible heat, latent heat, momentum, and carbon dioxide (CO_2_) fluxes. The mechanisms of pressure adjustment and vertical mixing that can make the MABL unstable were both identified. The WCE also acted to increase the surface winds and heat fluxes from the ocean to the atmosphere. Oceanic regions at middle and high latitudes are expected to absorb atmospheric CO_2_, and are thereby considered as sinks, due to their cold waters. Instead, the presence of this WCE in midlatitudes, surrounded by predominantly cold waters, caused the ocean to locally act as a CO_2_ source. The contribution to the atmosphere was estimated as 0.3 ± 0.04 mmol m^−2^ day^−1^, averaged over the sampling period. The CO_2_ transfer velocity coefficient (*K*) was determined using a quadratic fit and showed an adequate representation of ocean–atmosphere fluxes. The ocean–atmosphere CO_2_, momentum, and heat fluxes were each closely correlated with the SST. The increase of SST inside the WCE clearly resulted in larger magnitudes of all of the ocean–atmosphere fluxes studied here. This study adds to our understanding of how oceanic mesoscale structures, such as this WCE, affect the overlying atmosphere.

## Introduction

Sea surface temperature (SST) anomalies caused either by fronts or ocean eddies exert a crucial influence on surface winds and the marine atmospheric boundary layer (MABL) vertical structure that overlies them. Previous observational studies have investigated and diagnosed key mechanisms of ocean–atmosphere (OA) interactions in the Southwestern Atlantic Ocean (SWA), especially at the Brazil-Malvinas Confluence (BMC) region^1–6^. The BMC is recognized as one of the most energetic western boundary current regions in the global ocean^7^ and is formed by the confluence of the warmer and saltier waters of the Brazil Current (BC) with the colder and fresher waters from of Malvinas Current (MC).

In the BMC region, the water masses mixing from both the BC and MC define the western end of the subtropical convergence in the Southwestern Atlantic, a region known for the formation and subduction of South Atlantic Central Water (SACW). The latter spreads throughout the SWA into subsurface layers. The confluence generates strong lateral thermal gradients with values ranging from 0.01 °C km^−1^ up to 0.08 °C km^−1^ ^3^ and is highly variable in both time and space^8–11^ as it meanders around 38°S^12^. Also, the coastal region of the SWA is known to be one of the most important cyclogenesis regions of the atmosphere in the Southern Hemisphere (SH) with storm tracks eventually reaching the southern and southeastern parts of South America (SA)^13,14^. The SST gradient near the southeastern South American coast may play an essential role in cyclogenesis and cyclone intensification near 35°S^14^.

On the oceanic mesoscale, both the atmosphere and ocean are highly turbulent. In this context, ocean eddies occur as entities responsible for across-front mixing and transport of different physical, chemical, biological, and biogeophysical properties^15–18^, such as the mixing of tracers, kinetic energy, potential vorticity, phytoplankton concentration, and, particularly iron redistribution^15–17,19^. Among the factors producing ocean eddies in the BMC are the extreme thermal front that produces baroclinic instabilities^20^, the South American coastline orientation, and the ocean current reversals^15^ occurring when the eddies are shed from the main currents^8,15,21^. The BC reaches its southernmost positions during the austral spring and summer and the warm core eddies are shed at a yearly rate of seven or more^22^.

**Figure 1:**
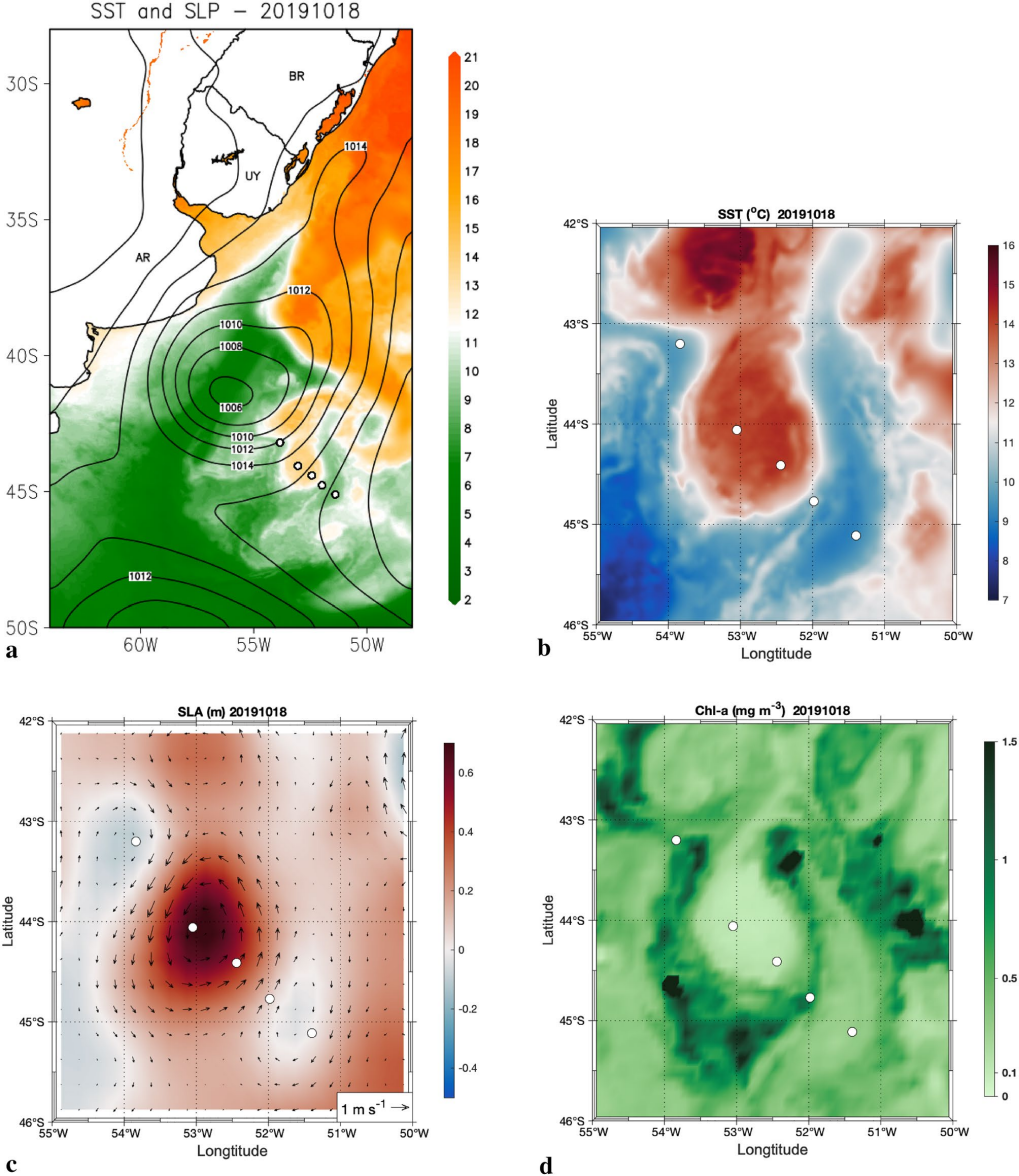
Images showing Southwestern Atlantic Ocean and study area. **(a)** MUR Sea Surface Temperature (°C) with Reanalysis ERA5 Sea Level Pressure (hPa) showing the study area in a broad view. **(b)** Same as **(a)**, but in view zoom of the eddy. **(c)** Sea Level Anomaly (m) relative to the geoid measured by Altimetry (colors) and derived absolute geostrophic velocity current vectors (m s^-1^). (d) Chlorophyll-a concentration (Chl-a in mg m^−3^). All data are for 18th October 2019. The white circles denote the Po/V *Almirante Maximiano* trajectory while crossing the eddy dipole and the XBTs and radiosondes launching positions. The symbols over the continent indicate the country names of Brazil (BR), Uruguay (UY) and Argentina (AR). Grid Analysis and Display System (GrADS), Version 2.2.1.oga.1. http://opengrads.org. MATLAB, Version 9.1.0.441655 (R2016b). https://www.mathworks.com.

Mesoscale ocean eddies tend to retain the properties of the original currents, thereby exhibiting different properties relative to their surroundings after shedding. They have characteristic time scales of months and spatial scales of several-tens to hundreds of kilometers^21–23^. They are also a source of intrinsic climate variability by modifying the large-scale circulation, SST, and ocean–atmosphere fluxes^6,24^. These eddies also have a large influence on biogeochemical cycles not only through lateral stirring and mixing^25^ but also through the vertical advection of nutrients in and around the eddies^26^, which are easily observed in chlorophyll-a images such as shown in Fig. 1c. Their surface thermal signature locally affects the overlying atmosphere, where warm (cold) eddies locally produce positive (negative) turbulent heat flux anomalies and an associated warm, well mixed, unstable (cool, stratified, stable) MABL^6,27,28^. These interactions feed back to affect the eddies themselves, since they also locally influence near-surface wind, cloud properties, and rainfall^29^.

Carbon dioxide (CO_2_) is one of the greenhouse gases present in the Earth's atmosphere and anthropogenic emissions have increased both atmospheric and oceanic concentrations, thus leading to climate change and to ocean acidification^30–32^. In general, tropical oceans are sources of CO_2_ to the atmosphere, while oceanic regions at mid to high latitudes absorb atmospheric CO_2_, thereby being considered as natural sinks of this gas^33^. One of the main sinks of CO_2_ is the ocean, known to absorb approximately one-third of the total anthropogenic emissions^34,35^. The Southern Ocean, which feeds the Malvinas Current, is an especially important sink of atmospheric CO_2_. Recent studies, however, reported uncertainties on ocean–atmosphere gas transfer velocity estimations^36^ and a decrease in this ocean’s absorption capacity due to increased wind intensity that modulates the CO_2_ ventilation from the deep ocean^37^. In addition, warm core eddies that travel to mid latitudes in the vicinity of subtropical oceanic fronts can play a role like the tropical ocean and act as a CO_2_ source to the atmosphere. This is the case we report here.

However, the physical mechanisms by which oceanic thermal signatures affect the stability of the atmosphere overlying eddies is still an active field of study. The study presented here sheds light on this topic by offering comprehensive results based upon rare, in situ observations of one such eddy. In particular, we discuss the ability of a warm core ocean eddy to modify the physical, dynamic, thermodynamic and CO_2_ properties of the oceanic environment where it lives, as well as its impacts on the overlying atmosphere. This type of phenomenon is still subsampled in this region. Our novel in situ and eddy-covariance turbulent flux data used in this study provides more understanding of the physical MABL stability mechanisms and ocean–atmosphere fluxes exchanges including momentum, heat, and CO_2_.

## Results

The Antarctic Modeling and Observation System (ATMOS) project, which is part of the Brazilian Antarctic Program (PROANTAR) sampled a WCE in the BMC region (Fig. 1a) during its first cruise named as ATMOS-1^38^. We used the Eddy Covariance (EC) method^39–43^ to measure the air-sea turbulent fluxes of CO_2_, momentum, sensible heat, and latent heat. To identify the impact of the eddy on its surrounding environment, such as the MABL dynamic and thermodynamic characteristics, we used complementary meteorological and oceanographic in situ data collected during the cruise.

### Atmospheric synoptic conditions

Our analysis begins by evaluating the large-scale atmospheric synoptic patterns that occurred during the ATMOS-1 cruise sampling period, from 18 to 19 October 2019, as detailed in Table 1. During most of the eddy-sampling period, the weather was cloudy, with light rain and fog. These atmospheric conditions were associated with the presence of an extratropical cyclone, which was migrating eastward and undergoing occlusion along the northern fringe of the WCE study area near the first westernmost sampling point (Fig. 1a). Around the time we launched our first radiosonde in this area, the sea level pressure in the central part of the extratropical cyclone was approximately 1006 hPa. The cyclone was located northwest of the launching position, while a zonally-oriented pressure ridge was situated to the south of the observational site. This synoptic configuration produced surface winds from the east-northeast during most of 18 October, with magnitude ranging from 5 to 18 m s^−1^ (Table 1). Winds with a northerly component promoting warm advection within the MABL were observed with the first two atmospheric soundings launched in the afternoon. From the evening of 18 October into the early morning of the following day, the wind direction in the MABL acquired a southerly component following the zonal displacement of the cyclone center towards the northeast of the study area. The cyclone continued to exhibit some deepening, with its central pressure dropping to 1002 hPa. In this final stage of the intensive observing period the surface wind speed varied from approximately 5 to 9 m s^−1^.

**Table 1:** Radiosonde date, time and position of launching. Sea surface temperature (SST), air temperature (T_air_), sea level pressure (SLP), relative humidity (RH), wind speed (WS), direction (WD). T_air_ and SLP were measured by ship’s automatic weather station (AWS).

Station	Date	Local time	Longitude (° W)	Latitude (° S)	SST (°C)	T_air_ (°C)		SLP (hPa)	RH (%)	WS (m s ^−1^)	WD (°)	MABL (m)
1	18/10/19	10:07	53° 50.57′	43° 12.20′	9.5	10.5		1010.3	95	17.9	79	960
2	18/10/19	16:21	53° 02.93′	44° 03.89′	14.4	11.0		1008.1	95	15.2	82	790
3	18/10/19	19:40	52° 26.37′	44° 24.40′	14.2	15.3		1008.9	95	10.4	154	980
4	18/10/19	23:36	51° 58.99′	44° 45.95′	9.0	13.0		1010.3	85	5.0	220	500
5	19/10/19	03:29	51° 24.14′	45° 06.43′	8.2	9.0		1010.3	85	9.0	202	750

SST that was obtained by the ship’s thermosalinographer and MABL top height that was estimated from radiosondes data.

### Oceanic synoptic physical and biological conditions

Satellite-derived Sea Level Anomaly (SLA), SST and chlorophyll-a (Chl-a) concentration data were used to identify the eddies present in the BMC region and define the ship route before the cruise in order to cross a pair of warm and cold core eddies as shown in Fig. 1. We clearly identified a well-defined warm core (anticyclonic) eddy, as are typically shed by the Brazil Current in the BMC (Fig. 1a). Southeast of it, a cyclonic cold core eddy (CCE) was also present (Fig. 1a,b). This eddy pairing suggests that it may be a dipole system. However, our main focus here is on the WCE, given its impressive signature and potential role in governing ocean–atmosphere interactions. The analysis of satellite-derived SST fields shows that the WCE central temperature is about 14 °C, decreasing to 9 °C along its edge. When reaching the edge of CCE via ship the SST was about 7 °C. This yields a SST difference of approximately 5 °C between the WCE and its border and of roughly 7 °C across the strong thermal front, similar to the ones seen previously at the BMC front^2,3^. This marked gradient is responsible for modifying the surrounding oceanic environment where this WCE is situated as we later demonstrate. The WCE had its center located at 44°S and 52°W with a mean radius of 95 km when observed. These characteristics are similar to a previous WCE analyzed in this region^28^. The structure extended north–south for 1.8° and east–west for 2°, as estimated using the SLA from satellite altimetry (Fig. 1d).

A direct relationship between SST, SLA, and the geostrophic currents derived from SLA can be seen in Fig. 1c. The WCE exhibited translational velocities reaching 1 m s^−1^ while the CCE velocities were less than 0.5 m s^−1^ (Fig. 1c). The lifecycle of a WCE detached from the Brazil Current can last for months with estimated translational velocities ranging from 5.8 to 7.8 km day^−1^ and, instead of just merging into surround waters by mixing or diffusion, can be re-assimilated by the parent current^21^. In our case, the eddy life cycle lasted 86 days (7 September 2019 to 1 December 2019) after which it was re-assimilated by the Brazil Current.

The WCE also imprinted a profound signal in the chlorophyll-a surface concentration field. The typical mean Chl-a values over the BC (MC) ranged from 0.015 to 0.5 (0.2 to 0.5) mg m^-3^ during September and October 2019^15^. In Fig. 1b,d it is evident that SST and Chl-a values at the WCE center are those typically found in BC waters. This confirms that this eddy was decoupled from the BC and transported its characteristics along its trajectory, a mechanism^44^ called eddy trapping. Furthermore, at the WCE periphery (where colder waters were located) we found higher Chl-a values, reaching 1.5 mg m^-3^. Similar Chl-a patterns in anticyclonic eddies in, and to the north of, the Southern Antarctic Circumpolar Current Front were previously observed^45^. It has also been observed that submesoscale density fronts (horizontal scale < 10 km) are commonly generated at the periphery of mesoscale eddies^44,46,47^. These submesoscale fronts are characterized by strong vertical ageostrophic circulation^48,49^, with upwelling rates reaching 10 m day^−1^ ^50–52^. Therefore, these regions are potentially an efficient route for vertical transport of nutrients^53^. Several studies based upon ocean color data support this idea, reporting high Chl-a surface concentration close to the periphery of eddies^45,47,54^, as also seen in our Fig. 1d.

In our case, the low Chl-a concentration in the WCE core may be due to eddy trapping during its formation, while the high Chl-a concentration at the borders might be explained by the action of submesoscale processes (Fig. 1d). Another region with high Chl-a concentration is located northeastward of the WCE, coinciding with the SST front location (Fig. 1b).

**Figure 2:**
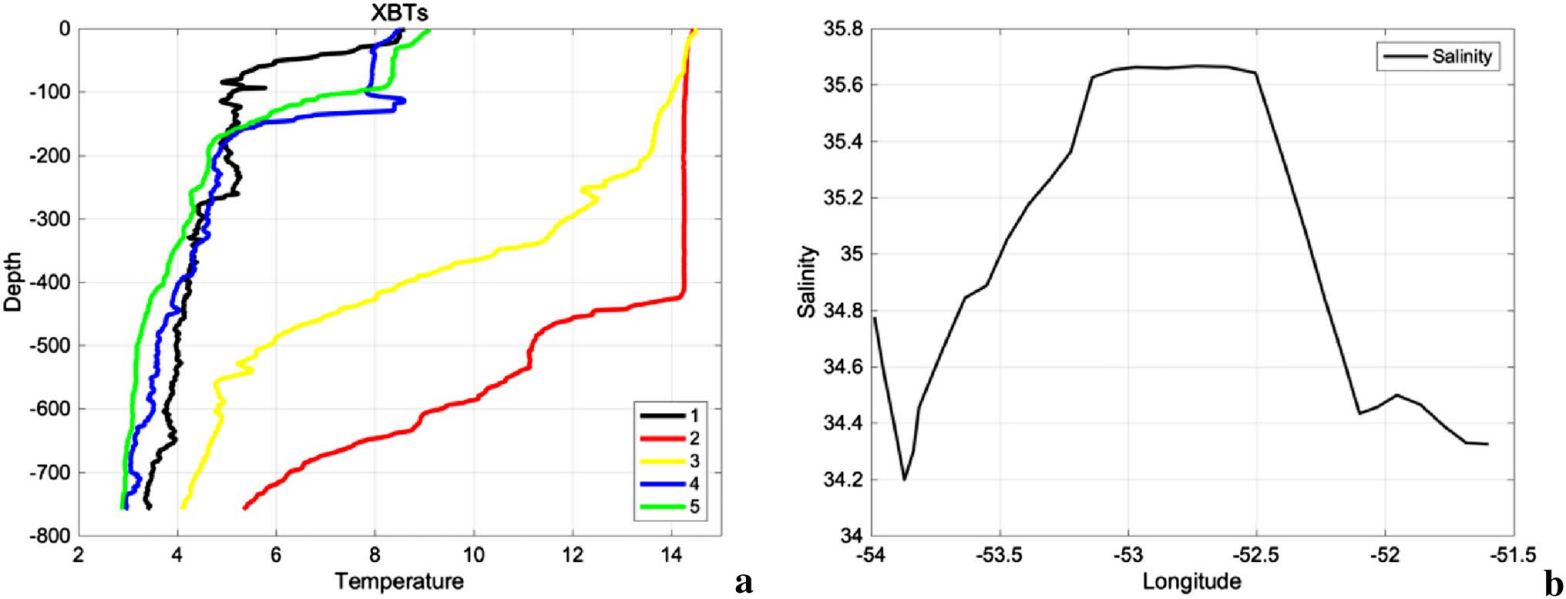
Synoptic, in situ measurements taken along Brazilian Navy Polar Vessel (Po/V) Almirante Maximiano (H-41) route while crossing the eddy. **(a)** XBT temperature (°C) depth profiles. The numbers in the legend denote de XBT positions, with 1 being the westernmost position and 5 being the easternmost position. The number 2 (red) is closest to the eddy core position. **(b)** Salinity, measured by ship thermosalinographer. MATLAB, Version 9.1.0.441655 (R2016b). https://www.mathworks.com.

Due to its anticyclonic circulation and consequent eddy-induced Ekman pumping, there is mass convergence inside the WCE that results in a deepening of the thermocline where the eddy is located (Fig. 2). An increase in Chl-a concentration (Fig. 1c) of 1.5 mg m^−3^ up to 4 mg m^−3^ at very localized spots is seen in the regions where the greatest SST gradients are located between the WCE and the cold waters around it (e.g. 44°S and 55°W). The same happens where the CCE is located (Fig. 1a), with a consequent resurgence of nutrient-rich colder waters. The high Chl-a concentration seen at the WCE periphery coincides with the climatological October-December Chl-a concentration^55^ of 1.5 mg m^−3^ associated with the Patagonian Shelf Large Marine Ecosystem (PSLME), one of the most productive and complex marine regions in the Southern Hemisphere^56^ and located slightly to the west of the WCE.

The vertical structure of our WCE clearly reveals a mixed layer depth of 426 m with temperatures ranging from 14 °C to 14.2 °C (red line in Fig. 2a). A well-stablished thermocline occurs below it, with temperatures ranging from 13.9 °C at 430 m to 5 °C at 760 m, as shown in Fig. 2a. Both indicate that this is a barotropic and well mixed temperature region in the ocean. The opposite is seen when moving from the eddy center towards its borders, where colder waters are present. This is seen mainly in the CCE (profile 4 and 5 in Fig. 2a). Those profiles reveal a shallower temperature mixed layer, reaching depths of approximately 100 m. Interestingly, the temperature-depth profile of station 4 (Fig. 2a) shows an inversion in the water temperature with respect to the depth near 106 m. There the water temperature decreased to 7.8 °C and below it increased to 8.6 °C at 116 m, and then continued to decrease downward as expected. This inversion can be associated with a subsurface meandering structure commonly present in oceanic, baroclinic frontal regions^57^. Below that we see a shallower and well-stablished thermocline, with an abrupt temperature decrease from 8.6 °C down to 4.8 °C at 192 m. Another important characteristic of the WCE is its surface salinity (Fig. 2b), with values ranging from about 34.2 at the borders to about 35.7 at the center of the eddy. This hat shape in the salinity surface profile is typical of warm core eddies in the BMC region, where lower salinity values are found at the eddy’s periphery where cold, less saline waters are present. The thermohaline values found inside our WCE confirm that it originated in a region of mixing between Tropical, Subantarctic, and South Atlantic Central Water^58^. Open questions persist about local processes such as eddy mixing, transport of tracers, and redistribution of other oceanic properties^59^. These questions mainly involve the specific theoretical processes and are dependent upon accurate vertical volume sampling of eddies^59^.

The WCE life cycle lasted for 86 days, estimated from a sequence of SLA satellite images. This eddy had dimensions of 2.20 10^5^ m in the meridional direction, 1.58 10^5^ m in the zonal direction and was approximately 350 m deep, with 15 °C average temperature, approximately, by the time it was sampled by the ship. Those dimensions indicate this WCE has a volume of 9.55 10^12^ m^3^, with approximately 5.59 10^19^ J of heat content excess compared to its surroundings. This heat content excess is larger compared to the few previous measurements made in the BMC region^11^. The net heat transfer from ocean to atmosphere over the eddy was estimated as 7.07 10^17^ J, considering that this excess of heat flux is a function of the eddy area during the sampling day. Our calculations are novel for this study region over this kind of oceanic mesoscale structures and reveal that approximately 1.3% of the ocean heat energy excess contained inside the WCE were transferred to the atmosphere, during the sampling period when our in situ measurements were made.

### Oceanic boundary layer and marine atmospheric boundary layer observations

**Figure 3:**
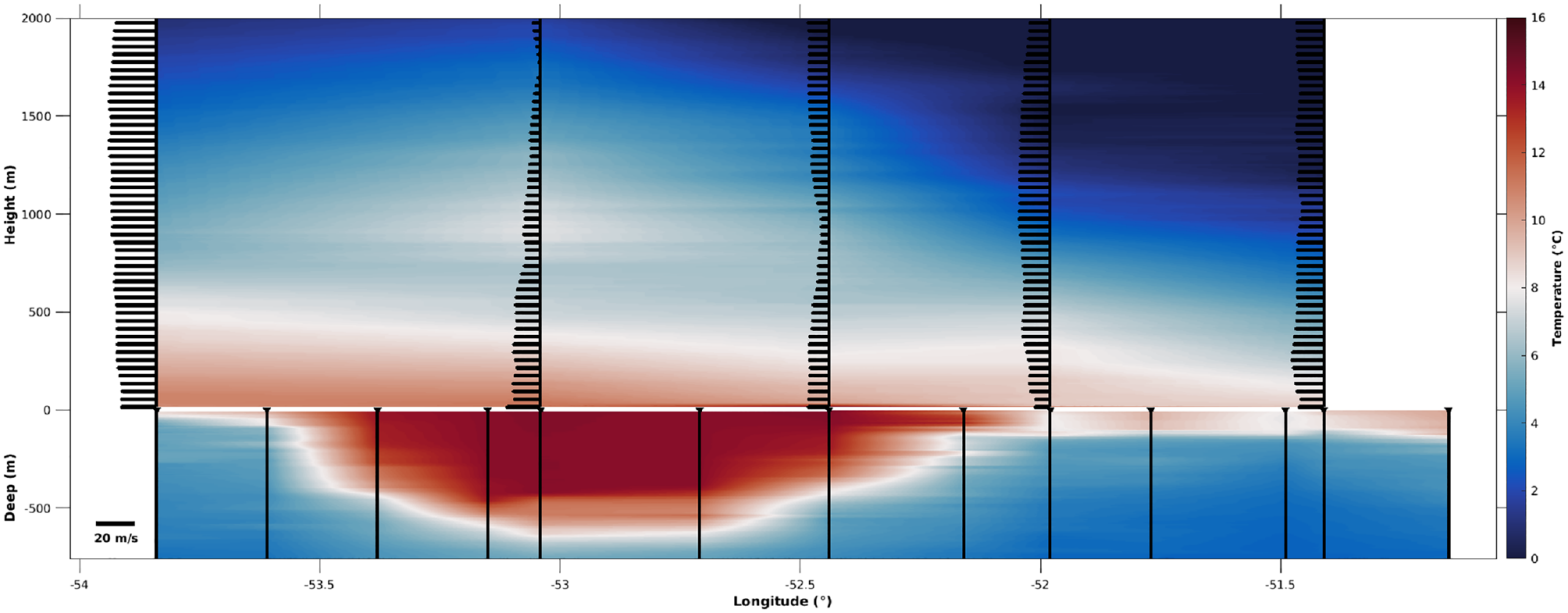
Temperature profiles (°C) of the atmosphere and ocean (colors) taken simultaneously by radiosondes and XBTs along the Brazilian Navy Polar Vessel (Po/V) Almirante Maximiano (H-41) route while crossing the eddy during 18^th^ October 2019. The lower part of this figure also displays the oceanic sounding positions. Wind magnitude (m s^-1^) in vectors is also displayed, superimposed on the air temperature. The vector size reflects the wind magnitude. MATLAB, Version 9.1.0.441655 (R2016b). https://www.mathworks.com.

The MABL and oceanic boundary layer (OBL) vertical profiles are shown in Fig. 3. The following analysis was made in order to evaluate the MABL static stability that is induced by the SST anomalies present in the ocean, as already described for the Eastern Equatorial Pacific^60^, the CBM^2,3^ and the SWA^40^. In the well-known vertical mixing mechanism^60^, the air buoyancy and turbulence intensity increases over warm waters. As a consequence, the MABL vertical wind shear is reduced, and stronger winds are generated at the sea surface. This process increases the transfer of momentum from the atmosphere to the ocean surface thus enhancing oceanic mixing processes and intensifying ocean–atmosphere fluxes^61^. An opposite situation is expected over cold waters. Figure 3 shows the MABL and OBL temperature vertical profiles (°C) taken along the Po/V Almirante Maximiano’s route during 18 October 2019. Wind magnitude vectors, overlaying the temperature profiles, clearly show that over warm waters the surface and near surface winds are stronger and present a small or non-existent vertical shear. This is a classic characteristic of a well-mixed and turbulent MABL, reflected also by the air temperature vertical homogeneity, as shown by the two westernmost atmospheric profiles in the upper half of Fig. 3. However, outside the WCE beyond its eastern border, the vertical wind shear increases, indicating an increase of the MABL stability and a decrease of surface wind magnitudes, as shown by the three easternmost atmospheric profiles in the upper half of Fig. 3. This process is part of the OBL and MABL interplay, where some of the surface oceanic characteristics are passed to the lower atmosphere. We need, however, to remark that our westernmost radiosonde (our first launching) does not show the expected typical behavior of cold waters locally modulating the MABL. We believe that this finding is associated with the influence of the extratropical cyclone previously described here.

### Heat fluxes and radiation balance

Next, we turn our attention to investigate the MABL stability using surface oceanographic and meteorological measurements in situ. The high-frequency sampling (20 Hz) made with our micrometeorological tower includes CO_2_ and water vapor (H_2_O) gas concentrations, three components of wind speed, air temperature (T_air_), barometric pressure, ship velocity, position and 3D angular accelerations and angular velocities. Short and long wave radiation measurements were acquired at lower frequency (1 acquisition per minute). SST and sea surface salinity (SSS) were taken from the ship’s thermosalinographer and hull’s ADCP. More details on the use of the instruments are shown in Table 2. The SST-T_air_ used here is one of the criteria for determining the stability of the MABL^2,3,40^ and provides an indication of the direction of heat fluxes typically showing positive (negative) values associated with positive (negative) fluxes from the ocean to the atmosphere^2,3,40^. All of our tower sensors were tested and calibrated by the Meteorological Instrumentation Laboratory of INPE before and after the experiment. Also, all of our measurements taken at high frequency, including sea level pressure, were in good agreement with the lower frequency data obtained from the ship automatic weather station (AWS), but not shown here.

**Table 2:** Meteorological and oceanic sensors installed on the micrometeorological tower and ship hull during the ATMOS-1 cruise.

Sensor	Model	Manufacturer	Variables sampled	Sampling rate (Hz)	Height/depth installation (m)
Integrated CO_2_/H_2_O Open-path gas analyzer and 3D sonic anemometer	IRGASON	Campbell Scientific	CO_2_ density, H_2_O density 3D wind components air temperature, air pressure	20	14.33
Net Radiometer	CNR2	Campbell Scientific	Net short and long wave radiation	1/60	12.87
Pyranometer	CMP3-L	Kipp & Zonen	Incoming short wave radiation	1/60	15.26
Compass	C100	KVH Industries	Direction	20	15.2
GPS	GPS16X-HVS	Garmin	Position	20	15.13
Multi axis inertial sensing system	MotionPak II	Systron Donner Inertial	3D accelerations and 3D angular velocities	20	14.14
Barometric Pressure Sensor	CS106	Vaisala	Air pressure	1/60	15
Thermosalinograph	SBE45	Sea Bird	SSS and SST	1/60	− 5

**Figure 4:**
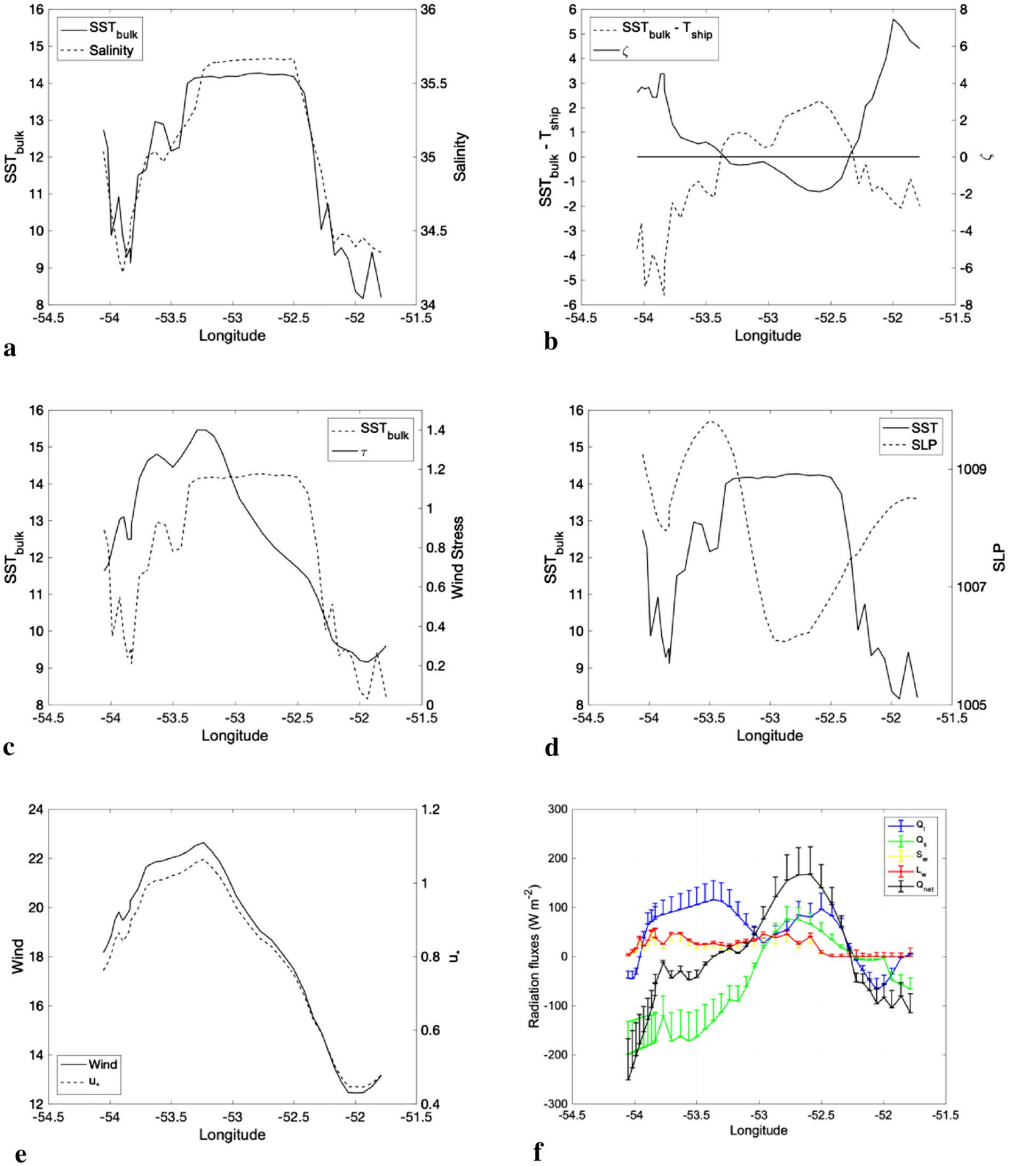
Synoptic, in situ measurements taken along Brazilian Navy Polar Vessel (Po/V) Almirante Maximiano (H41) route while crossing the eddy. **(a)** SST_bulk_ (°C) and salinity. **(b)** Stability parameters, $\zeta$ and SST—T_air_ (°C). **(c)** SST_bulk_ (°C) and wind stress (N m^-2^). **(d)** SST_bulk_(°C) and sea level pressure (hPa). **(e)** Wind speed magnitude and friction velocity (u_*_), both in m s^-1^. **(f)** Components of net heat flux (Q_net_), short and long wave radiation (S_w_ and L_w_), latent and sensible heat fluxes (Q_l_ and Q_s_, both measured by eddy covariance) in W m^-2^. The bars in **(f)** are the standard error oriented up for visual clarity representing 95% confidence interval. However, they must be interpreted both up and down. All information is derived from the ship-borne meteorological data. MATLAB, Version 9.1.0.441655 (R2016b). https://www.mathworks.com.

The measurements clearly show that the WCE exerts a marked presence by modifying the surrounding waters and providing a large source of heat to the atmosphere, as seen in Fig. 4. The SST at the eddy core was 14 °C (Fig. 4a), which was 2 °C higher than T_air_ taken on the ship's bow tower at 16 m height from the sea surface^62^ and measured at the same times and locations. This is quantified by the strong vertical thermal difference (SST-T_air_) seen on the time series in Fig. 4b.

Positive values of SST-T_air_ define an unstable MABL and the larger this difference is, the more unstable the MABL is. A second MABL stability parameter evaluated here is the Monin–Obukhov stability parameter ($\zeta$), shown in Fig. 4b. This parameter tends to corroborate our SST-T_air_ series, with negative values over the region that SST-T_air_ is positive. The parameter $\zeta$ is a function of the scaling parameter L defined as the Obukhov length and indicating the height of the boundary layer where the buoyancy factors dominate compared to the turbulent vertical transport caused by the wind^63^. Negative values of $\zeta$ indicate a MABL that is statically unstable while positive values mean statically stable conditions.

**Figure 5:**
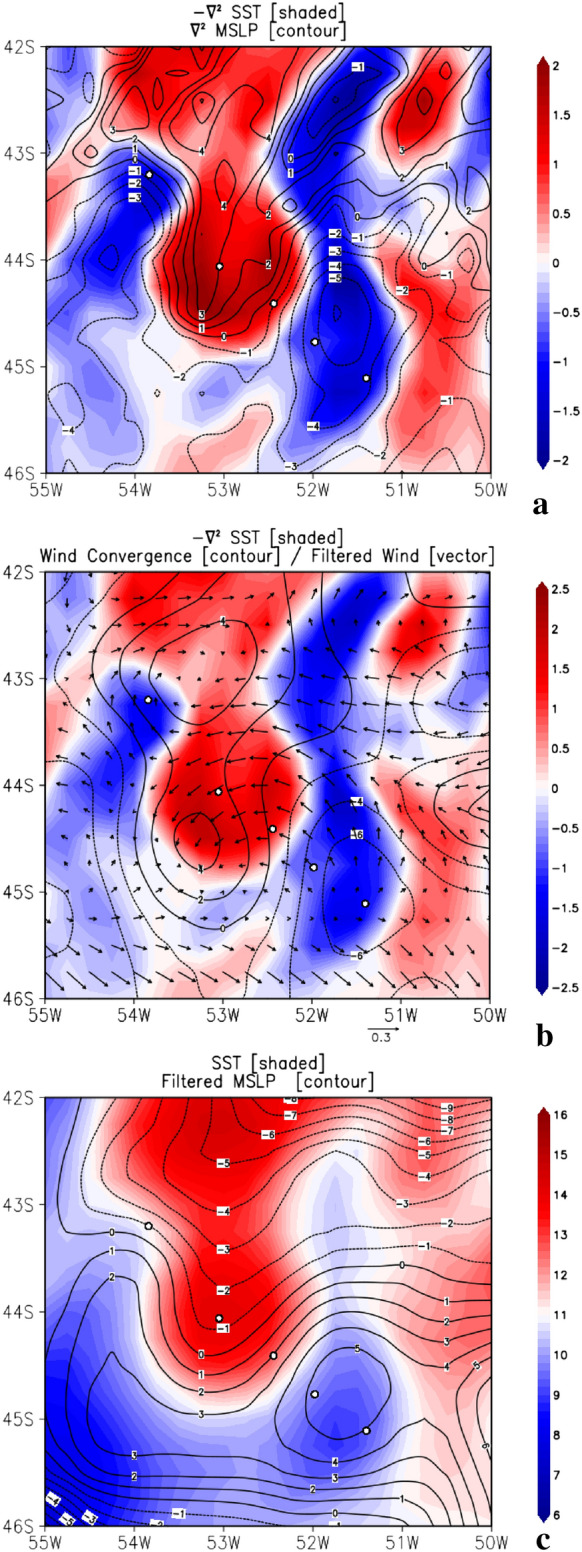
Maps of 10-day-averaged surface atmospheric and oceanic variables from ECMWF ERA5 reanalysis. a) negative Laplacian of Sea Surface Temperature (− ${\nabla }^{2}$ SST 10^−9^ K m^-2^) is shaded and Sea Level Pressure (${\nabla }^{2}$ SLP 10^−9^ Pa m^-2^) is contoured. **(b)** Laplacian of Sea Surface Temperature (- ${\nabla }^{2}$ SST 10^−9^ K m^-2^) is shaded, wind convergence ($\nabla \mathrm{w}$ 10^–6^ s^-1^) is contoured and high-pass-filtered field of wind (vectors). c) Sea Surface Temperature (°C) is shaded and high-pass-filtered field of Sea Level Pressure (hPa) is contoured. Grid Analysis and Display System (GrADS), Version 2.2.1.oga.1. http://opengrads.org.

Strong surface wind speed was observed on the westernmost side of the WCE, reaching a maximum value over the warmer waters of the WCE core (Fig. 4e). Wind speed minima are observed over the cold waters along the eastern side of the WCE, at the CCE center. It is interesting to note that the WCE advects and retains the BC thermohaline properties, showing higher SSS values of 35.6. These positive values of SST-T_air_ (Fig. 4b) and higher SSS values (Fig. 4a) span a considerable area of the WCE’s surface. Lower SLP values coincided with higher SST values (Fig. 4d), demonstrating that the lower atmosphere was influenced by the WCE's local modulation. In order to substantiate this, we complement our study using the ERA5 reanalysis (Fig. 5). Discussion follows in the last paragraph of this section.

This WCE role as a heat source to the atmosphere is also clearly noticed in the heat balance presented in Fig. 4f,based on our in situ observations. The spatio-temporal variability of net heat flux (Q_net_) indicates the WCE net heat contribution, where positive values mean the WCE induces a heat flux directed from the ocean to the atmosphere. The turbulent heat fluxes are proportional to the temperature and specific humidity differences between the air and the sea surface, as well as to the wind magnitude and the stability coefficient. The positive contribution of the sensible (Q_s_) and latent (Q_l_) heat fluxes estimated by the Eddy Covariance (EC) method and the thermal longwave radiation (L_w_) are also seen over most of the area of the WCE. As previously described, during the whole sampling period the sky was cloudy and this is reflected in the relatively low diurnal net short wave radiation (S_w_) fluxes. The mean EC heat fluxes are also corroborated by the bulk calculation of fluxes^64^, as can be verified in the supplementary material presented in Figure A1.

The impressive case of eddy-induced MABL modulation presented here using observational data was also verified by an independent reanalysis data. The independent ERA5 SST data, although having lower resolution in respect to satellite estimates (Figure A2a), clearly showed a well-defined pattern associated with our eddy as also seen in Fig. 1. The reanalysis wind stress ($\tau$) overlays SST in that figure to show that the wind magnitude increases over warmer waters. Moreover, when $\tau$ components are filtered and the higher frequency modes are retained and displayed (Supplementary Figure A2b), the effect of the wind acceleration (deceleration) over warmer (colder) waters is highlighted even more, corroborating what was already reported for other regions of the world ocean^25^. The atmospheric surface modulation by the WCE is strong enough to make its effects noticed in higher levels of the MABL column of air overlying it (Supplementary Figure A2a and A2b). The ascendant air movement is coincident with the region where higher SST values are located and a higher MABL top occurs. Conversely, descendant air movement is noticed where lower SST values and MABL top heights occur. These results, together with our in situ atmospheric vertical profiles (Fig. 3) clearly demonstrate the capacity of the WCE to influence the MABL vertical structure. Reanalysis data also showed that the MABL height approximately varies from 650 m over warmer waters to 450 over colder waters. However, the reanalysis underestimates the MABL height, compared to what was estimated through radiosondes. The height was approximately 960 m, 790 m and 980 m over warmer waters and 500 m and 750 m over colder waters (Table 1).

ERA5 data were also used to investigate the role of WCE on the local modulation of the overlying atmosphere. Besides the vertical mixing mechanism^60^ already explored earlier in this study, another mechanism can explain the surface wind modulation by SST. This mechanism occurs in regions of strong SST gradients and is known as the pressure adjustment mechanism^65^. It relates the Laplacian of the SLP (${\nabla }^{2}$ SLP) and SST with the reversed sign (−${\nabla }^{2}$ SST) with the surface wind convergence^66^. In this way, it is possible to isolate the WCE effects on the MABL modulation from background effects caused by the large-scale atmospheric systems. During the WCE life cycle, we observed that for the 10-day period ranging from 15 to 25 October 2019 the eddy remained almost stationary in the location where it was sampled by the ship during the ATMOS-1 campaign. We used this period to calculate the mean fields of SST, SLP, and wind magnitude at 10 m and then estimate the − ${\nabla }^{2}$SST, ${\nabla }^{2}$ SLP and wind convergence fields (Fig. 5a,b). Figure 5b also shows the filtered wind field at 10 m. There we can see that the wind diverges in regions with lower SST and converges in regions with higher SST. Furthermore, we observed positive (negative) values of ${\nabla }^{2}$ SLP in regions with positive (negative) values of − ${\nabla }^{2}$SST (Fig. 5a). This relationship indicates that the surface wind convergence occurring over the WCE (Fig. 5b) is associated with the pressure adjustment mechanism induced by the SLP gradient, observed between the WCE region (lower SLP) and the neighboring regions (cold waters, higher SLP—Fig. 5c). Note that in our case a geographical shift is observed in the convergence area with respect to the eddy center, which is a feature that was also observed in similar studies^67^.

### Carbon dioxide analysis

**Figure 6:**
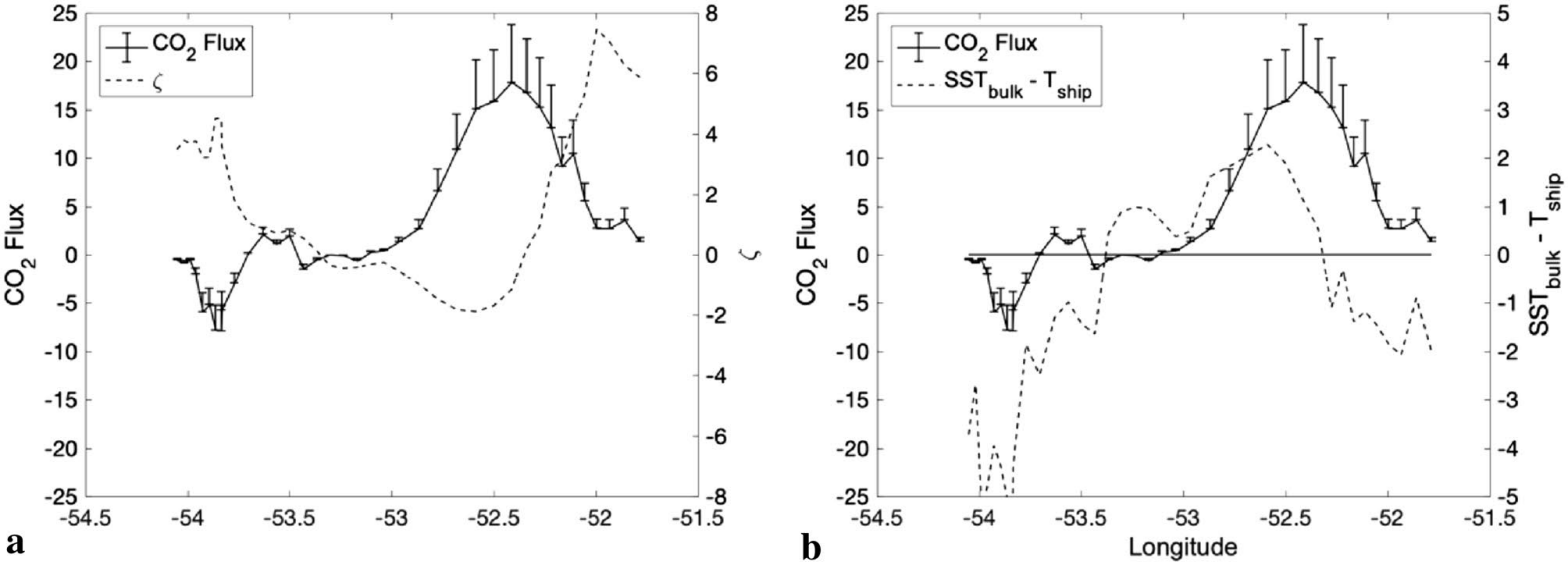
Atmospheric in situ CO_2_ fluxes measured by Eddy Covariance method along Brazilian Navy Polar Vessel (Po/V) Almirante Maximiano (H-41) route while crossing the eddy. **(a)** CO_2_ fluxes ($\upmu$mol m^-2^ s^-1^) and stability parameter $\zeta$ 10^2^. **(b)** CO_2_ fluxes ($\upmu$mol m^-2^ s^-1^) and stability parameter SST—T_air_ (°C). The error bars are the standard error and are oriented up. The bars are the standard error oriented up for visual clarity representing 95% confidence interval. However, they must be interpreted both up and down. MATLAB, Version 9.1.0.441655 (R2016b). https://www.mathworks.com.

We finish our data analysis using our high-frequency data to show how our WCE effectively modified the surrounding ocean–atmosphere CO_2_ fluxes. To our knowledge this kind of in situ observation is unique in the Southwestern Atlantic Ocean. This region supports one of the largest CO_2_ sinks of the global ocean. Previous studies^68^ reveal that the region has an annual average, ocean–atmosphere difference of the CO_2_ partial pressure (ΔpCO_2_) of − 31 atm$\mu$. The average CO_2_ ocean–atmosphere flux is − 3.7 mol$\mathrm{m}$ m^−2^ day^−1^ (negative indicates a sink where the ocean absorbs CO_2_ from the atmosphere). However, the Southwestern Atlantic is a transition region to the Southern Ocean, where many warm eddies are shed from the BC to colder waters with the ability to change the environment they transit by carrying physical, chemical and biological characteristics from their region of origin^28,59,61^. Our measurements displayed in Fig. 5 clearly show that the analyzed WCE carries the original Brazil Current characteristics further southwards than this current alone is capable of doing. The waters inside the eddy are warmer (Fig. 1a), saltier (Fig. 2b) and depleted of nutrients (Fig. 1d). During the ATMOS-1 campaign, the wind direction (Table 1) varied from northeast to southwest (first and third quadrants) causing different atmospheric advection conditions. The ocean–atmosphere CO_2_ fluxes measured during the field campaign tend to reflect this environment and follow the MABL stability variability. The parameter $\zeta$ and values of SST-T_air_ provided indications of how turbulent the atmospheric layer was near the ocean surface. There is a sign change of these parameters due to cold advection associated with both cyclone transition and the reduction of SST at the end of the ship's transect during ATMOS-1. As a result, positive and lower CO_2_ fluxes were then observed. This is also corroborated by comparing our CO_2_ flux measurements with both atmospheric stability parameters $\zeta$ and SST-T_air_, shown in Fig. 6. Ocean–atmosphere CO_2_ fluxes were positive (from the ocean to the atmosphere) in the region of larger SST anomalies and MABL instability, where $\zeta$ was negative (Fig. 6a) and SST-T_air_ (Fig. 6b) and − ${\nabla }^{2}$ SST (Fig. 4f) were both positive. In conclusion, the effect of the WCE studied here was to modify the typical behavior of the Southwestern Atlantic Ocean, an ocean expected to be a CO_2_ sink.

**Figure 7:**
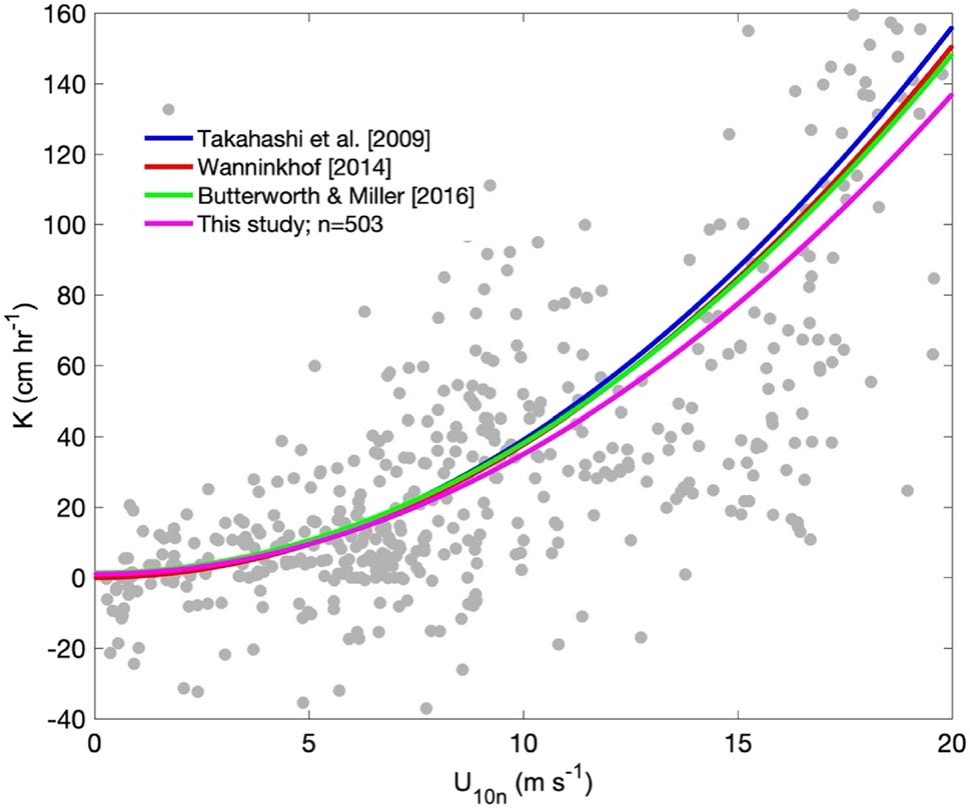
Relationship between the CO_2_ transfer velocity coefficient and the neutral wind speed at 10 m calculated from the data collected in this experiment. The quadratic fitted curve *K* = 0.34.U_10n_^2^ – 0.32 U_10n_ + 0.94 with r^2^ = 0.75 is represented by the magenta line. The blue, red and green curves represent the CO_2_ transfer coefficient obtained in the literature^36,69,70^. MATLAB, Version 9.1.0.441655 (R2016b). https://www.mathworks.com.

In order to assess the quality of the CO_2_ fluxes calculated in this study, the CO_2_ ocean–atmosphere transfer velocity coefficient (*K*) was computed and compared to some classic values found in the literature^69,70^ and with a more recent one developed for the Southern Ocean^36^ (Fig. 7). We found a quadratic adjustment (*K* = 0.34 U_10n_^2^ – 0.32 U_10n_ + 0.94) between the CO_2_ transfer coefficients and the neutral wind speed collected at 10 m (U_10n_) during the cruise. For U_10n_ less than 7 m s^−1^ our curve showed a good agreement with previous studies. However, for U_10n_ greater than 7 m s^−1^, the *K* values were lower than the curves used for comparison. Even so, it is possible to observe that the *K* curve was able to satisfactorily represent the expected behavior. When the wind speed is zero then *K* = 0.94 cm h^−1^, which is higher than the studies used here for comparison. We can associate this discrepancy with processes such as internal turbulence at the ocean surface^36^ or the biological activity that is characteristic of this region.

## Discussion

In summary, ocean eddies play a fundamental role in transporting and mixing properties between regions with heterogeneous characteristics. In this observational turbulent flux study in the Southwestern Atlantic Ocean, we presented and highlighted the ability of a warm core ocean eddy shed from the Brazil Current to modify both the ocean and the surrounding atmosphere. Since 2012 the Southwestern Atlantic Ocean has been sampled during research cruises using the Eddy Covariance (EC) method to directly measure the ocean–atmosphere heat, momentum, and gas fluxes in combination with more traditional methods of observing the ocean and the atmosphere from ships^2,3,40^. The present study shows that the lateral SST gradients produced by the presence of a WCE in cold waters extensively affect the MABL stability and that the eddy effects may cross the top of the MABL and reach the troposphere (Supplementary Figure A3).

There is a lively debate about the mechanisms by which the atmosphere near the ocean surface may become unstable in various regions of the world^3,61,66,71–73^. The pressure adjustment mechanism, explained above, is not easy to identify using observational studies due to the sparse resolution that is often intrinsic to the data set. However, the good spatio-temporal quality of our observational data and the support of complementary (ERA5) reanalysis data allowed depiction of the effectiveness of the pressure adjustment mechanism^66,74^. The mechanism causes a wind convergence over warm-core eddies and a wind divergence over cold-core eddies, measured through the link between the SST to the SLP Laplacian fields. Concomitantly, the stability parameters determined from the ocean–atmosphere temperature difference and the Monin–Obukhov stability parameter ($\zeta$) together diagnose a MABL static stability induced by the SST anomalies^2,3,40,60^. The increased (diminished) vertical mixing is associated with a more unstable (stable) MABL over warmer (colder) waters. These sudden changes in the SST and the increase (decrease) of vertical turbulent mixing related to the large (small) ocean–atmosphere temperature differences establish a decreased (increased) atmospheric vertical wind shear.

The EC is considered the best method to quantify the ocean–atmosphere CO_2_ fluxes because its uncertainties are of the order of only 5%^75^. These uncertainties are much smaller than those associated with the bulk methods that use (uncertain) transfer coefficients. From this technique, our direct unprecedented CO_2_ measurements indicate an eddy contribution of 0.3 + /− 0.04 mol$\mathrm{m}$ m^−2^ day^−1^ to the atmosphere over the ATMOS-1 sampling period. If one considers its entire life cycle of about three months, then the amount of CO_2_ that may be transferred to the atmosphere can reach values of 25.8 ± 3.56 mmol m^-2^. The ocean–atmosphere CO_2_ transfer velocity coefficient, computed with our data and quadratically fitted to wind speed, yielded good performance by agreeing with *K* determined in other CO_2_ studies^36,69,70^, as shown in Fig. 7. Warm core eddies commonly found in the Southwestern Atlantic Ocean, like the one studied here, therefore are important natural contributors to the atmospheric carbon budget throughout their respective life cycles.

This study increases our understanding of how a mesoscale warm core ocean eddy affects its surrounding environment. We conclude that the particular eddy studied here actively modified both the physical and the CO_2_ exchanges between the ocean and the atmosphere in the Southwestern Atlantic Ocean. This indicates a need for further investigating the effect of the overall eddy “population” over time as they affect the atmosphere overlying the Southwestern Atlantic and throughout the world ocean.

### Data and methodology

All in situ data were collected on board the Brazilian Navy Polar Vessel (Po/V) *Almirante Maximiano* (H-41) during the ATMOS-1 cruise. This paper presents and discusses these novel and independent high-frequency measurements of heat, momentum, and CO_2_ fluxes taken onboard the ship.

The study region is located in the Southwestern Atlantic Ocean near the BMC region. During the period from 18 to 19 October 2019, the ship crossed a train of both warm and cold core eddies (Fig. 1). While crossing the study region, many oceanographic and meteorological observations were performed. During the ATMOS-1 cruise, a total of six Expendable Bathy-Thermographs (XBTs) were deployed at the same locations where six radiosondes were launched (Table 1). The oceanic sampling was complemented by 8 CTD (Conductivity, Temperature, Depth) stations. Unfortunately, the CTD castings were not synchronized in time with the atmospheric measurements; instead, they were performed during the two following days, when the ship crossed back along the same trajectory. The reason for that was to minimize the effects of possible changes in the large-scale atmospheric synoptic patterns on modifying our in situ data due to non-local effects. Recall that we aimed primarily to use the ocean–atmosphere measurements made on the WCE to investigate its potential to locally change the atmosphere immediately above it (Fig. 3). The applied methodology here is similar to that used in our previous work^2,3,40^. At port, a micrometeorological tower was installed on the bow of Po/V *Almirante Maximiano* following previous methodology^40–43^ with different sensors able to collect ocean–atmosphere turbulent flux data of momentum, latent and sensible heat, CO_2_ and water vapor. This is based on Eddy Covariance (EC) methodology and used a sampling frequency of 20 Hz in order to obtain 30-min averaged fluxes^40–43^. Surface radiation data were also collected using the micrometeorological tower for computing ocean–atmosphere radiation fluxes. All data presented in Figs. 4 and 6 were resampled to 30-min intervals. All oceanographic and meteorological sensors and main characteristics of use are shown in Table 2. The net surface heat flux (${Q}_{net}$) was obtained using the previously computed ocean–atmosphere heat fluxes and the radiation components using the expression:

1 $${Q}_{net}={S}_{w}+ {L}_{w}+{Q}_{l}+{Q}_{s}$$

where ${S}_{w}$ is the net shortwave radiation, ${L}_{w}$ is the net longwave radiation, ${Q}_{l}$ and ${Q}_{s}$ are latent and sensible heat fluxes, respectively.

The ocean–atmosphere CO_2_ flux estimates presented here refer to the total emission of the WCE during the period of its sampling (Fig. 5). It represents 12 h of sampling summing up to 157.2 mol$\upmu$ m^−2^ s^−1^. When extrapolated to a daily emission, we arrive at 0.3 + /- 0.04 mol$\mathrm{m}$ m^−2^ day^−1^. This same estimate was then multiplied by the estimated life of this eddy (86 days) resulting in a total of 25.8 + /− 3.56 mol$\mathrm{m}$ m^−2^. We regard this estimate as, to our knowledge, the first approximation ever made to represent the total emission of CO_2_ produced by a warm core eddy into the atmosphere in the Southwestern Atlantic Ocean during a typical eddy life span.

The ocean–atmosphere transfer velocity of CO_2_ is obtained through the relationship between variables of these two environments according to the expression FCO_2_ = *K* Sco_2_ ΔpCO_2_. Where F*CO*_*2*_ is the CO_2_ flux (mmol m^−2^ day^−1^) obtained from the EC, *K* (cm^−1^) is the gas transfer velocity coefficient and is directly related to the wind speed^69,70^ and was adjusted to Schmidt's number of 660. *Sco*_*2*_ (mol m^−3^ atm^−1^) is the CO_2_ solubility coefficient (Weiss, 1974) in seawater, using the sea temperature and salinity sampled by the ship’s thermosalinographer that was available. The ΔpCO_2_ (μatm) is the difference between the partial pressure of CO_2_ between the ocean (*p*CO_2w_) and the atmosphere (*p*CO_2a_). The *p*CO_2w_ was obtained from the climatological fields^69^ and *p*CO_2a_ from a LI 7000 closed-path CO_2_ analyzer installed at the ship’s bow. A total number of 503 (out of 1473) CO_2_ flux intervals were use in the *K* calculation, after an EC quality control procedure. These data were obtained during 31 days of cruise in the Southwest Atlantic region and in the Southern Ocean from 3 transits of the Drake Passage between October 6th and December 2nd. Using these data, the quadratic equation *K* = 0.34.U_10n_^2^ – 0.32 U_10n_ + 0.94 with r^2^ = 0.75 that describes the relationship between *K* and the neutral wind speed at 10 m (U_10N_) was found.

Satellite data were also used in this study. The Group for High Resolution Sea Surface Temperature (GHRSST) Level 4 analysis derived from the Multi-scale Ultra-high Resolution (MUR) sensor was used. This is a merged, multi-sensor satellite and in situ SST analysis product with spatial resolution of 0.01° latitude/longitude and daily temporal resolution provided by the Jet Propulsion Laboratory (http://podaac.jpl.nasa.gov). The sea level anomaly (SLA) used here is the sea surface height above or below the mean sea surface height relative to the period of 1993 to 2012. These are daily data based on multi-mission altimeter satellite gridded SLA product, and distributed at level 4, 0.25º latitude/longitude resolution by the European Copernicus Marine Environment Monitoring Service (http://marine.copernicus.eu). The surface geostrophic currents are also derived from this same data set.

Our in situ data analysis was complemented with the European Centre for Medium-Range Weather Forecasts (ECMWF) reanalysis data set ERA5 (http://www.ecmwf.int). ERA5 represents the newest version of hourly estimates of a large number of atmospheric, land, and oceanic climate variables. The surface (pressure level) data covers the globe with 0.1º (0.25º) latitude/longitude horizontal resolution. The reanalysis is catalogued on 37 pressure levels in the vertical. Using the laws of physics by means of a 4-D variational data assimilation technique a vast number of observations are combined with model outputs.

In order to retain the smaller-scale signal contained in ERA5 data, we smooth the variable fields using a successive moving window (spatial) filter with 3 × 3 grid point size that is subtracted from the total field. Many studies have used space–time filters for this purpose^24,76,77^. Our choice, although a simplified spatial filtering strategy yielded in consistent results since we were able to see in our maps the expected spatial coincidence between the mesoscale SST features present in our study area and the wind stress (Figure A2b), the surface wind at 10 m height (Fig. 5b), and SLP (Fig. 5c). The same filtering technique was applied on the vertical profiles shown in supplementary Figure A3.

The calculation of the heat content in the eddy is an important estimate because it allows us to know how much heat is transported by the eddy during its transit. In this case, the properties originating in the Brazil Current end up being transported southwards to the Subtropical Front. This heat energy is available for both contributing to interior ocean processes, such as water mass mixing, and modifying the lower atmosphere. However, the eddy volume, which is the basic measure to all later estimates, is not easy to be precisely calculated. Here we assumed an ellipsoid format for our WCE^11^. We determined the structure’s mean diameter in the zonal and meridional directions from the SLA satellite image of 18 October 2019. The WCE surface area can then be calculated as:

2 $${A_e} = \left( {{\raise0.7ex\hbox{${{d_x}}$} \!\mathord{\left/ {\vphantom {{{d_x}} 2}}\right.\kern-\nulldelimiterspace} \!\lower0.7ex\hbox{$2$}}} \right) \cdot \left( {{\raise0.7ex\hbox{${{d_y}}$} \!\mathord{\left/ {\vphantom {{{d_y}} 2}}\right.\kern-\nulldelimiterspace} \!\lower0.7ex\hbox{$2$}}} \right) \cdot \pi$$

where ${A}_{e}$ is the eddy area, and ${d}_{x}$ = 158 × 10^3^ m and ${d}_{y}$ = 220 × 10^3^ m are the eddy’s zonal and meridional diameters, respectively $.$

Using our in situ XBT data, we estimated a mean depth of 350 m for the WCE. The eddy volume (${V}_{e}$) is then obtained as follow:

3 $${V}_{e}={A}_{e} .{ d}_{e}$$

The $${d}_{e}$$ is mean depth of the WCE. The eddy heat content ($${OHC}_{e})$$ is then obtained by:

4 $${OHC}_{e}=\rho .{ c}_{p} . {V}_{e}.({T}_{w}-{T}_{c})$$

where $\rho$ is the mean water density inside the eddy, ${c}_{p}$ is the specific heat capacity of the water at the sea surface, and ${V}_{e}$ is the eddy’s volume. ${T}_{w}$ and ${T}_{c}$ (K) are the mean warmer water surface temperature inside the WCE and colder water temperature outside the eddy, respectively. This method of calculation allows us to quantify the WCE heat content excess compared to its surroundings, termed here as ${OHC}_{e}$.

A similar calculation was performed aiming to obtain the integrated excess of heat transferred from ocean to atmosphere, which is not trivial since the determination of the height at which the heat fluxes approach zero above the surface boundary layer (SBL) remains a key problem^78,79^. This methodology was previously used for estimates made at fixed locations over land^79^. In our case, however, the ship observations were performed with both time and space varying. As a consequence, the result cannot be the heat flux, but rather the heat excess transferred from the eddy surface to the atmosphere during the ATMOS-1 cruise^78^. The net heat energy transferred from the WCE to the atmosphere is the difference between the measurements performed over the warm (in Eq. 5, ${Q}_{net\_w})$ and cold water (in Eq. 5, ${Q}_{net\_c})$. Those estimates were obtained from Eq. 1 and chosen from Fig. 4f, where ${Q}_{net\_w}$= 160 W m^-2^ and ${Q}_{net\_c}$= − 100 W m^-2^.

5 $${Tot}_{e}=\left({Q}_{net\_w}-{Q}_{net\_c}\right).{A}_{e}. {E}_{t}$$

and

6 $${HE}_{net}=\left({Tot}_{e}.100\right)/{OHC}_{e}$$

where ${Tot}_{e}$ is the net heat energy available for transfer from the WCE to the atmosphere. ${E}_{t}$ is assumed to be 1 day. Finally, we calculated ${HE}_{net}$ as a fraction of net energy heat transferred to the atmosphere.

## Supplementary Information

Supplementary Figures.
