# Publisher Correction: Oceanic eddy-induced modifications to air–sea heat and CO_2_ fluxes in the Brazil-Malvinas Confluence

**DOI:** 10.1038/s41598-021-93956-5

**Published:** 2021-07-12

**Authors:** Luciano P. Pezzi, Ronald B. de Souza, Marcelo F. Santini, Arthur J. Miller, Jonas T. Carvalho, Claudia K. Parise, Mario F. Quadro, Eliana B. Rosa, Flavio Justino, Ueslei A. Sutil, Mylene J. Cabrera, Alexander V. Babanin, Joey Voermans, Ernani L. Nascimento, Rita C. M. Alves, Gabriel B. Munchow, Joel Rubert

**Affiliations:** 1 Laboratory of Ocean and Atmosphere Studies (LOA), Earth Observation and Geoinformatics Division (OBT), National Institute for Space Research (INPE), São José dos Campos, SP Brazil; 2 Earth System Numerical Modeling Division, Center for Weather Forecast and Climate Studies (CPTEC), National Institute for Space Research (INPE), Cachoeira Paulista, SP Brazil; 3 Scripps Institution of Oceanography, University of California, San Diego, La Jolla, CA USA; 4 Federal University of Maranhão, São Luís, MA Brazil; 5 Federal Institute of Education, Science and Technology of Santa Catarina, Florianópolis, SC Brazil; 6 Agricultural Engineering Department, Federal University of Viçosa, Viçosa, MG Brazil; 7 Department of Infrastructure Engineering, University of Melbourne, Victoria, Australia; 8 Atmospheric Modeling Group (GruMA), Department of Physics, Federal University of Santa Maria, Santa Maria, RS Brazil; 9 Federal University of Rio Grande Do Sul, Porto Alegre, RS Brazil; 10 Southern Space Coordination (COESU), National Institute for Space Research (CRS/INPE), Santa Maria, RS Brazil

Correction to: *Scientific Reports* 10.1038/s41598-021-89985-9, published online 20 May 2021

The Supplementary Information file published with this Article contained an error in its file extension.

This error has now been corrected in the Supplementary Information file that accompanies the original Article.

