# Supplementary material for: Oceanic eddy-induced modifications to air–sea heat and CO2 fluxes in the Brazil-Malvinas Confluence
Source: Sci Rep. 2021 May 20;11:10648. doi: 10.1038/s41598-021-89985-9 (PMC8137957; doi:10.1038/s41598-021-89985-9)
Supplement: Supplementary file 1 — Supplementary Figures. [file 41598_2021_89985_MOESM1_ESM.pdf]

Oceanic eddy-induced modifications to air-sea heat and CO_2 fluxes in the Brazil-Malvinas Confluence

Luciano P. Pezzi[1*], Ronald B. de Souza[2], Marcelo F. Santini[1], Arthur J. Miller[3], Jonas T. Carvalho[1], Claudia K. Parise[4], Mario F. Quadro[5], Eliana B. Rosa[1], Flavio Justino[6], Ueslei A. Sutil[1], Mylene J. Cabrera[1], Alexander V. Babanin[7], Joey Voermans[7], Ernani L. Nascimento[8], Rita C. M. Alves[9], Gabriel B. Munchow[9], Joel Rubert[10].

1- Laboratory of Ocean and Atmosphere Studies (LOA), Earth Observation and Geoinformatics Division (OBT), National Institute for Space Research (INPE), São José dos Campos, SP, Brazil.
2- Earth System Numerical Modeling Division, National Institute for Space Research (INPE), Cachoeira Paulista, SP, Brazil.
3- Scripps Institution of Oceanography, University of California, San Diego, La Jolla, CA, USA.
4- Federal University of Maranhão, São Luís, MA, Brazil.
5- Federal Institute of Education, Science and Technology of Santa Catarina, SC, Brazil.
6- Agricultural Engineering Department, Federal University of Viçosa, Viçosa, MG, Brazil.
7- Department of Infrastructure Engineering, University of Melbourne, Victoria, Australia.
8- Atmospheric Modeling Group (GruMA), Department of Physics, Federal University of Santa Maria, Santa Maria, RS, Brazil.
9- Federal University of Rio Grande do Sul, Porto Alegre, RS, Brazil
10- Southern Space Coordination (COESU), National Institute for Space Research (INPE), Santa Maria, RS, Brazil

[*] L. P. Pezzi, luciano.pezzi@inpe.br

Supplementary material

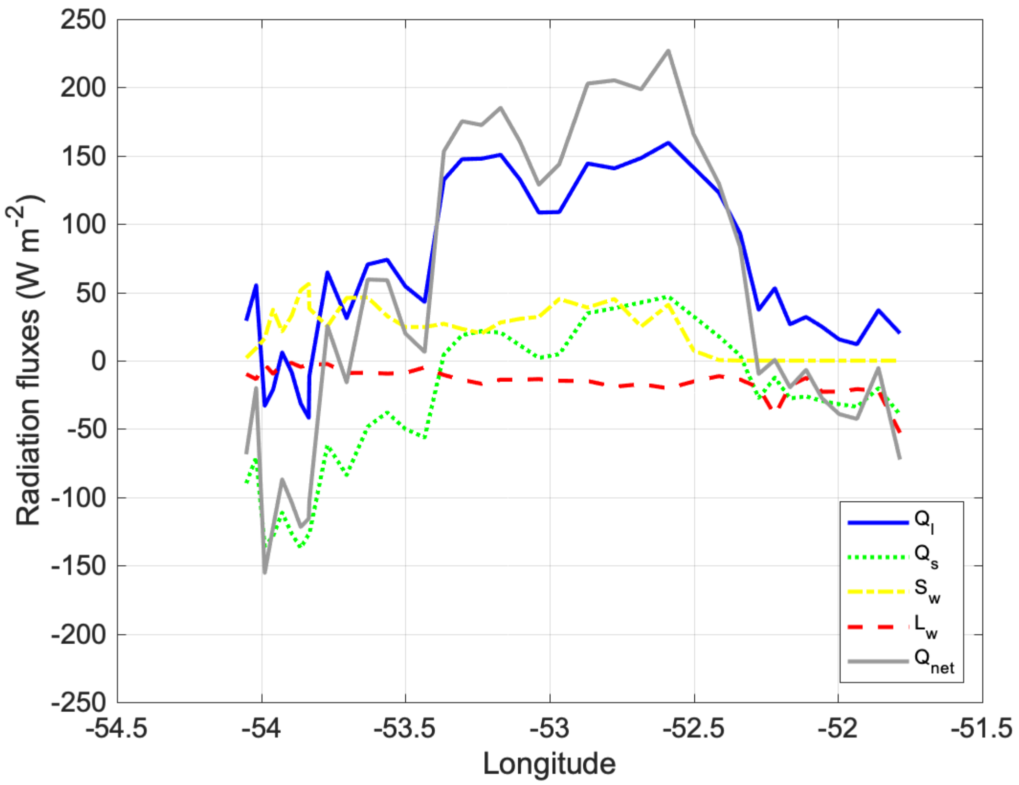

A1) Heat balance calculation made along Brazilian Navy Polar Vessel (Po/V) Almirante Maximiano (H-41) route while crossing the eddy. The heat balance components are net heat flux (Q_{net}), short and long wave radiation (S_w and L_w), latent and sensible heat fluxes (Q_l and Q_s) in (W m^{-2}). This panel brings heat balance components derived from bulk flux parametrization (Edson et al. 2013). MATLAB, Version 9.1.0.441655 (R2016b). https://www.mathworks.com

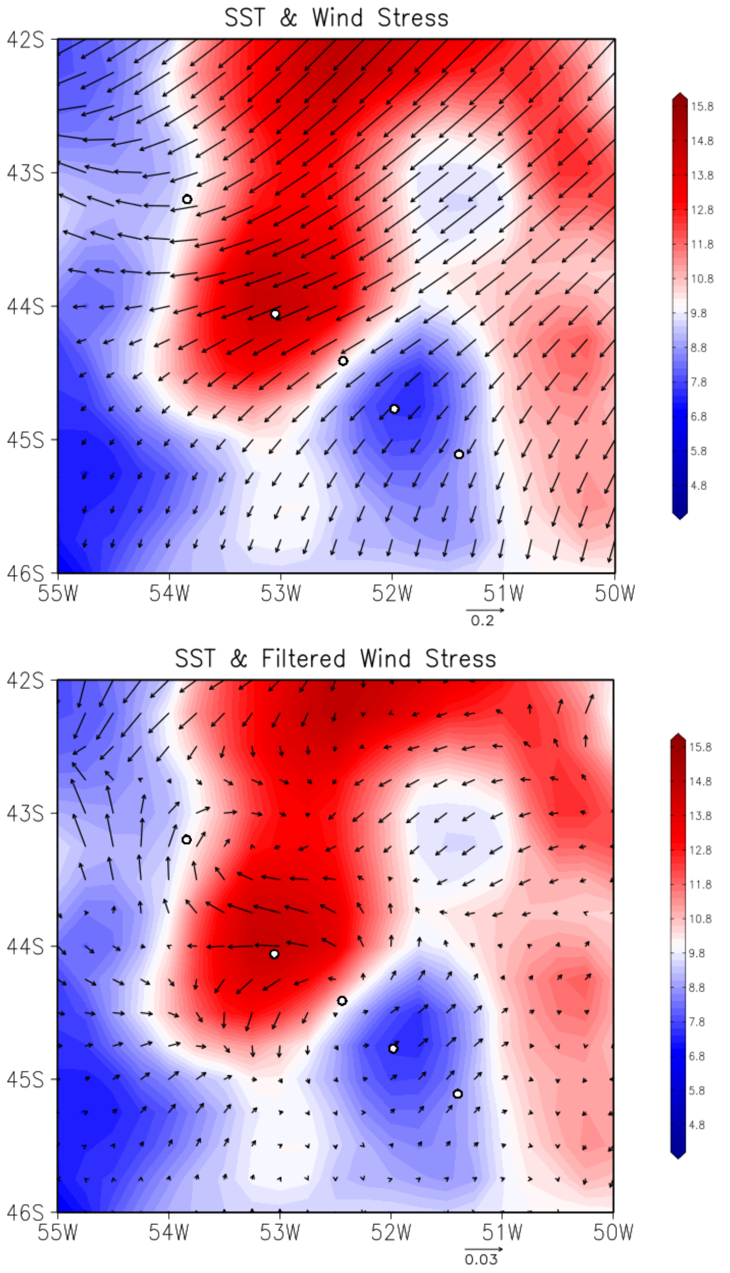

a)

b)

A2) Wind Stress and SST (a) Filtered Wind Stress and SST (b). All variables are 3 hours data averaged over the eddy sampling period that started at the 18th and finished 19th October 2019 from ERA 5 reanalysis. Grid Analysis and Display System (GrADS), Version 2.2.1.oga.1. http://opengrads.org

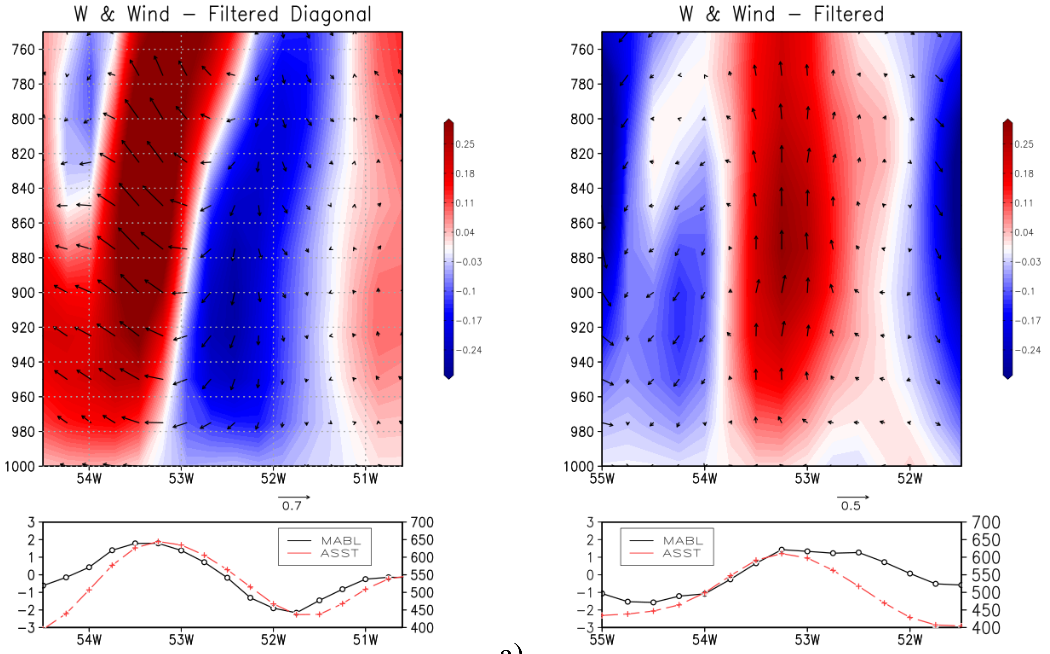

a) b)

A3) Vertical profile of vertical wind (m.s^{-1}), magnitude in colors with direction superimposed (relative to zonal wind, u) in vectors (upper panel) and filtered SST (ASST) in °C (red line, left axis) and Marine Atmospheric Boundary Layer (MABL) top in meters (black line, right axis). In the upper panel u was scaled (u/5) in order to compare and plot the vectors with w. a) Filtered wind along the Brazilian Navy Polar Vessel (Po/V) Almirante Maximiano (H-41) route while crossing the eddy. Refer to Figure 1 to see the H-41 route. b) Filtered wind along 43.5 °S. All variables are 3 hours data averaged over the eddy sampling period that started at the 18th and finished 19th October 2019, from ERA 5 reanalyzes. Grid Analysis and Display System (GrADS), Version 2.2.1.oga.1. http://opengrads.org
